# Association of *IL-1β*, *IL-6*, *TNF-α*, and *TGFβ1* Gene Polymorphisms with Recurrent Spontaneous Abortion in Polycystic Ovary Syndrome

**DOI:** 10.1155/2020/6076274

**Published:** 2020-05-05

**Authors:** Afrah F. Alkhuriji, Suliman Y. Al Omar, Zainb A. Babay, Manal F. El-khadragy, Lamjed A. Mansour, Wazirah G. Alharbi, Mahmoud I. Khalil

**Affiliations:** ^1^Department of Zoology, College of Science, King Saud University, Riyadh, Saudi Arabia; ^2^Department of Obstetrics and Gynaecology, King Saud University, King Khalid University Hospital, Riyadh, Saudi Arabia; ^3^Department of Biology, Faculty of Science, Princess Nourah Bint Abdulrahman University, Saudi Arabia; ^4^Department of Zoology and Entomology, College of Science, Helwan University, Cairo, Egypt; ^5^Faculty of Sciences of Gabès, University of Gabès, Erriadh City 6072, Zrig Gabès, Tunisia; ^6^Department of Biological Sciences, Faculty of Science, Beirut Arab University, Lebanon; ^7^Molecular Biology Unit, Department of Zoology, Faculty of Science, Alexandria University, Egypt

## Abstract

Recurrent spontaneous abortion (RSA) is a common pregnancy-associated complication of polycystic ovary syndrome (PCOS) which is an endocrine malfunction disease. Patients with PCOS may have several underlying contributing and interrelated factors, which have been reported in women with RSA. The incidence rate between PCOS and RSA remains uncertain. The aim of this study is to determine the possible association of *IL-1β*-511C/T, *IL-6*-174G/C, *TNF-α*-1031T/C, and *TGFβ1*-509T/C with RSA patients with or without PCOS. A total of 140 RSA patients, 70 of which were PCOS patients, and 140 healthy females with no history of RSA or PCOS were included in this study. PCR amplification, genotyping, and sequence analysis were employed to investigate the presence of the polymorphisms. The genotypic and allelic frequencies were calculated separately for each subject. Out of the four studied polymorphisms, the *IL-1β*-511C/T genotype in RSA without PCOS patients (12.7%) was significantly different compared with that in control subjects (*p* = 0.047). For *IL-6*-174C/G, there was a tendency towards more CC carriers among RSA with PCOS patients (10%) than in controls (3%). The GG genotype in RSA women with PCOS (60%) was significantly different compared with that in control subjects (*p* = 0.033), and the GC genotype in RSA with PCOS patients (30%) showed a marginal significant difference compared with that in control subjects (*p* = 0.050). Significant difference was identified in the allelic frequencies in RSA patients with PCOS compared to controls (*p* = 0.025). *IL-6*-174G/C and *TNF-α*-1031T/C polymorphisms are significantly associated with RSA patients in Saudi patients with PCOS, while the *IL-1β*-511C/T polymorphism is significantly associated with RSA patients without PCOS.

## 1. Introduction

Recurrent spontaneous abortion (RSA) is a common pregnancy-associated complication. It is defined as three or more consecutive pregnancy losses that occur prior to 24 gestational weeks at a rate of 15-20% and affecting about 2-4% of couples [1, 2]. The causes for RSA are associated with untreated hypothyroidism, uterine anatomic irregularities, parental chromosomal abnormalities, and uncontrolled diabetes mellitus. Other likely or possible etiologies include thrombophilias, immunological abnormalities, endocrine issues, obesity, infections, parental age, and environmental factors. Around half of all cases remain unexplained [3, 4].

Polycystic ovary syndrome (PCOS) is an endocrine malfunction disease, characterized by chronic anovulation and hyperandrogenism [5]. The definition of the syndrome has been much debated. In 2003, a joint European Society of Human Reproduction and Embryology and American Society for Reproductive Medicine (ESHRE/ASRM) consensus meeting produced a refined definition of PCOS, namely, the presence of two out of three criteria: (1) oligo- and/or anovulation; (2) hyperandrogenism (clinical and/or biochemical); and (3) polycystic ovaries, with the exclusion of other etiologies [6, 7]. PCOS affects 4–18% of reproductive-age women, depending on the diagnostic criteria used [8, 9]. Other clinical presentations of PCOS include obesity, cystic ovaries, insulin resistance, hirsutism, and coronary heart disease [10]. PCOS is a familial polygenic condition attributed to both genetic and environmental factors [11]. Mutations, polymorphisms, and differential regulation of genes may contribute to the pathogenesis of PCOS [10].

Disruption in the communication between mother and fetus, via the placenta and decidua, can result in pregnancy loss via spontaneous RSA [12, 13]. Patients with PCOS may have several underlying contributing and interrelated factors, which have been reported in women with RSA. These factors include hyperinsulinaemia, hyperhomocysteinaemia, hyperandrogenaemia, insulin resistance, obesity, and poor endometrial receptivity [14, 15]. RSA association with PCOS, which occurs in 50% of total pregnancies, is a frequent obstetric complication [16]. The prevalence of PCOS in RSA women remains highly uncertain. Once pregnant, it is not clear how PCOS leads to RSA [12]. Surprisingly, there is an extremely wide variation in results; between 4.8% and 82% of women with RSA were found to have PCOS on ultrasound [17, 18]. PCOS is associated with various metabolic and reproductive dysfunctions [19–21]. Elevated numbers of circulating inflammatory mediators in response to noxious stimuli have been observed in PCOS [19, 22].

Cytokines are the cell signaling molecules that play a pivotal role in pregnancy as immune regulators, mediating gametogenesis, implantation, and fetal development [19]. Many mutations and polymorphisms in genes coding for cytokines have been associated with either RSA or PCOS [23–28]. Interleukin-1 beta (IL-1*β*) plays a significant role in reproductive physiology and has been implicated in ovulation, fertilization, and embryo implantation as a critical regulatory factor [29]. Interleukin-6 (IL-6) is a multifunctional cytokine involved in regulation of immune responses and inflammation. IL-6 promotes B-cell differentiation and T-cell proliferation [30]. Tumor necrosis factor-alpha (TNF-*α*) is a proinflammatory cytokine produced by different immune cells including antigen stimulated T cells, lymphocytes, and NK cells [31]. Transforming growth factor-beta (TGF-*β*) is a pleiotropic cytokine that functions in tissue fibrosis, wound healing, and embryonic development [32]. Studies on the association of cytokine polymorphisms with either RSA or PCOS reveal inconsistent results. To this aim, we studied the polymorphisms *IL-1β*-511C/T (rs16944), *IL-6*-174G/C (rs1800795), *TNF-α*-1031T/C (rs1799964), and *TGFβ1*-509T/C (rs1800469) to assess the association of these polymorphisms in RSA patients with or without PCOS.

## 2. Materials and Methods

### 2.1. Sample Collection

The study was approved by the Medical Ethics Committee of King Khalid University Hospital, Riyadh, Saudi Arabia (approval code E-10-132). All patients were requested to sign an informed consent form in order to participate.

### 2.2. Subjects

The study was conducted on 280 Saudi women (age 18–45 years) who were divided into three groups. Group 1: seventy Saudi women with RSA attending the outpatient clinics for abortion at the Department of Obstetrics and Gynaecology at the King Khalid University Hospital, Riyadh, or other hospitals, consecutively referred, were included in the study. Inclusion criteria were females with a history of at least three RSA episodes. Women with RSA in whom the causes of abortion were known were excluded by performing anatomical, hormonal, and chromosomal tests. Infection tests for toxoplasma, cytomegalovirus, rubella virus, hepatitis B and C viruses, and human immunodeficiency virus (HIV) were carried out on these females. On the other hand, women with RSA with autoimmune causes, including anticardiolipin antibody, were also excluded from the study. Group 2: seventy Saudi women with RSA attending the outpatient clinics for abortion at the Department of Obstetrics and Gynaecology at the King Khalid University Hospital, Riyadh, or other hospitals, consecutively referred, were included in the study. Blood samples from Saudi women were collected from January 2018 to July 2018. Inclusion criteria were females with PCOS and a history of at least three RSA episodes. Group 3: one-hundred forty healthy Saudi females with at least two healthy children and no history of RSA or PCOS were recruited from King Khalid University Hospital. The protocol of this investigation was approval by the Medical Ethics Committee of King Khalid Hospital. All women were requested to sign the informed consent in order to participate.

### 2.3. DNA Extraction

EDTA tubes (BD Vacutainer, USA) were used for collecting blood samples by venepuncture. Genomic DNA was extracted from peripheral blood using the Puregene DNA purification kit (Qiagen, Germany) according to manufacturer instructions. The concentration and purity of each DNA sample were determined using the NanoDrop 2000 Spectrophotometer (Thermo Fisher Scientific, USA). The genomic DNA was stored at -20°C until use.

### 2.4. PCR Amplification and Genotyping

Genotyping of *IL-1β*-511C/T (rs16944), *IL-6*-174G/C (rs1800795), *TNF-α*-1031T/C (rs1799964), and *TGFβ1*-509T/C (rs1800469) was performed using assay-on-demand TaqMan® SNP genotyping assays according to the manufacturer's instructions (Applied Biosystems, USA). Genotyping was done by the allelic discrimination method (VIC- and FAM-labelled primers). Assay-on-demand TaqMan assays were ordered from Applied Biosystems: Assay ID C___1839943_10 (rs16944), C___1839697_20 (rs1800795), C___7514871_10 (rs1799964), and C___8708473_10 (rs1800469). The reaction was performed in 10 *μ*l volume using a ViiA™7 Real-Time PCR, per manufacturer's instructions (Applied Biosystems, USA). Analyses of amplification products were performed using ViiA™7, ver 1.1. (Applied Biosystems, USA). All the experimental conditions are available on demand.

### 2.5. Statistical Analysis

Results are reported as mean value ± standard error of the mean (SEM). The Student *t*-test was employed to compare the demographic data between patients and control subjects. The observed and expected numbers of each genotype of each polymorphism for patients and control subjects were compared in Hardy-Weinberg equilibrium by using a chi-squared (*χ*^2^) test. A chi-squared test (*χ*^2^) and logistic regression were used to examine the effects of genetic polymorphisms on RSA and PCOS. The criterion of the association is using odds ratio (OR) and 95% confidence interval (CI). A replicated stratification analysis was utilized in order to ensure that there are no confounding effects for age, body mass index (BMI), height, and weight. All statistical analyses were conducted using IBM-SPSS (version 22). *p* < 0.05 was considered statistically significant.

## 3. Results

The demographic data of patients and controls are shown in Table 1. Body mass index (BMI) showed a significant difference between patients and controls (*p* = 0.022). The analysis of the Hardy-Weinberg equilibrium on patients and control subjects revealed that any SNPs did not deviate from the Hardy-Weinberg equilibrium (Table 2).

The analysis of genotype and allele distribution of *IL-1β*-511C/T (rs16944), *IL-6*-174G/C (rs1800795), *TNF-α*-1031T/C (rs1799964), and *TGFβ1*-509T/C (rs1800469) gene polymorphisms was performed on all subjects (Table 3). The *IL-1β*-511*TT* genotype in RSA patients without PCOS was significantly different when compared with that in healthy controls (*p* = 0.047). For *IL-6*-174G/C, there were more *CC* carriers among RSA patients with PCOS (10%) than in controls (3%). The *GG* genotype in RSA women with PCOS was significantly different compared with that in control subjects (*p* = 0.033), and the *GC* genotype in RSA with PCOS patients showed a marginal significant difference compared with that in control subjects (*p* = 0.050). Although the genotypes of *TNF-α*-1031T/C did not differ significantly between the RSA patients with or without PCOS and healthy controls, significant difference was identified in the allelic frequency in RSA patients with PCOS compared to controls (*p* = 0.025). The genotype and allele frequencies of *TGFβ1*-509T/C did not differ significantly between the RSA patients with or without PCOS and healthy controls.

## 4. Discussion

Cytokines are important for normal pregnancy development, and any abnormality in quantity or locality of expression may affect trophoblast-endometrial interaction leading to pregnancy complications including RSA [33, 34]. Although the contribution of a broad spectrum of SNPs in cytokine-coding genes to RSA has been extensively investigated, their role remains unclear [28, 31, 34–36]. We examined the possible associations of *IL-1β*, *IL-6*, *TNF-α*, and *TGFβ1* gene polymorphisms with RSA Saudi patients with or without PCOS.

IL-1 system has a pivotal role during early pregnancy, and the elevated levels of IL-1*β* increase the probability of successful and complete implantation [28, 37]. In this study, we investigated the *IL-1β*-511C/T polymorphism in the promoter region in Saudi female patients (RSA with or without PCOS and controls). The results showed a significant difference in the *IL-1β*-511*TT* genotype in Saudi female patients (RSA without PCOS) as reported previously [38, 39]. The *IL-1β*-511C/T polymorphism may be a predisposing factor for RSA susceptibility and was shown to be associated with RSA in Korean women [40]. In a meta-analysis retrospective case-control study, *IL-1β*-511C/T did not reveal any significant association with the risk of RSA in North Indian women [41]. The same polymorphism has been associated with PCOS in Chinese women [42] and not in Caucasian and Indian women [19, 43]. Contrary to our results, in Iranian Azeri women with RSA, *IL-1β*-511C/T polymorphism may not be involved [44]. Inflammatory factors IL-1*β* and IL-1ra had correlation with obesity of PCOS patients; PCOS patients who carried T allele of *IL-1β* gene promoter region (-511) and V allele of *IL-1ra* gene were at high risk of obesity [45]. These alleles might be the genetic basis of the rising of IL-1*β* and IL-1ra levels in blood serum of PCOS patients and are associated with the infertility occurrence of PCOS patients [46].

Here, the results showed no significant differences in the frequency of the *IL-6*-174G/C polymorphism between RSA patients with or without PCOS and controls, which is contradicting to previously published data [13, 47]. Along with other reported results, our results exhibited significant difference in the *GG* genotype frequency of *IL-6*-174G/C in RSA patients with PCOS and controls [27, 48–50]. *IL-6* promoter region polymorphism may be related to metabolic abnormalities seen in PCOS [50]. However, *IL-6*-174G/C was not associated with the presence of PCOS but appears to be associated with the clinical characteristics of PCOS women [51].

Certain polymorphisms in the *TNF-α* gene have been associated with altered TNF-*α* secretion and are linked with pregnancy complications [31]. TNF-*α* genetic polymorphisms might be a risk factor for RSA [52]. Here, the results showed a significant difference in the allele frequencies of *TNF-α*-1031T/C in RSA patients with PCOS. A strong association was reported previously in Korean, South Indian, and Chinese Han populations [53–55]. Other studies reported significant association of *TNF-α*-1031T/C genotype frequency in RSA patients without PCOS [56, 57].

The level of TGF*β*1 was shown to increase in PCOS patients' sera and ovaries compared to non-PCOS women [58–60]. TGF-*β* regulatory pathway appears to play a critical role in PCOS development and may be an important therapeutic target in patients with PCOS [61]. The increased TGF*β*1 bioavailability due to increased TGF*β*1 and decreased levels of its receptor may contribute to PCOS pathogenesis and ovarian hyperstimulation [60, 62]. Several *TGFβ1* gene polymorphisms have been reported; some have been shown to have an important correlation with TGF*β*1 production and disease severity in studies involving the association between *TGFβ1* polymorphisms and RSA [63]. The current study has shown no significant allele or genotype associations of *TGFβ1*-509T/C in RSA patients with or without PCOS confirming data previously published [63, 64]. *TGFβ1* gene single nucleotide polymorphisms (SNPs) and haplotypes were associated with PCOS in Chinese women [65]. Out of four studied SNPs of the *TGFβ1* gene, the frequencies of *TGFβ1*-509T/C allele negativities and CC genotypes showed positive associations with PCOS [60].

IL-1, IL-6, and TNF-*α* play pivotal roles in reproductive physiology, including follicular maturation, ovulation, and implantation; these are parameters that are all affected in PCOS patients [66, 67]. Although a meta-analysis study suggested positive relationships between the *TNF-α*-1031T/C and *IL-6*-174G/C polymorphisms and PCOS risk, there were no associations between *IL-1β*-511C/T polymorphism and PCOS risk [48]. In another study, the results of a meta-analysis suggest that the *IL-1β*-511C/T and *IL-6*-174G/C polymorphisms may not be associated with PCOS risk [67]. Most of the studies that occurred in Asia reported the association of *IL-1β*-511C/T, *TNF-α*-1031T/C, and *IL-6*-174G/C with PCOS susceptibility development. Nevertheless, further investigations based on genome-wide association studies and cytokine gene SNPs are needed to better characterize PCOS risk factors [27].

## 5. Conclusions

In conclusion, the inconsistency in reported studies may be attributed to the selection criteria and ethnic background for studied subjects. In Saudi women, *IL-6*-174G/C and *TNF-α*-1031T/C polymorphisms are associated with RSA patients with PCOS, while *IL-1β*-511C/T polymorphism is associated with RSA patients without PCOS.

## Figures and Tables

**Table 1:** Demographic data of patients and control subjects.

Study group	RSA with PCOS (*n* = 70)	RSA without PCOS (*n* = 70)	Control (*n* = 140)	*p*	*F*
Age (yr)	33.28 ± 0.56	33.85 ± 0.71	32.94 ± 0.57	0.597	0.517
Height (cm)	157.52 ± 0.77	157.37 ± 0.98	158.38 ± 0.46	0.474	0.749
Weight (kg)	72.62 ± 1.65	75.34 ± 2.23	70.19 ± 1.23	0.075	2.617
BMI (kg/m^2^)	29.22 ± 0.61	30.35 ± 0.79	28.01 ± 0.50	0.022	3.860

Values are means ± standard error of the mean (SEM). RSA = recurrent spontaneous abortion; PCOS = polycystic ovary syndrome; BMI = body mass index; *p* = significance; *F* = one-way analysis of variance (one-way ANOVA).

**Table 2:** Observed and expected value of each gene polymorphism in RSA patients with or without PCOS and controls by the Hardy-Weinberg equilibrium.

Gene polymorphism		RSA with PCOS			RSA without PCOS			Control		
		*Observed*	*Expected*	*p*	*Observed*	*Expected*	*p*	*Observed*	*Expected*	*p*
*IL-1β*-511C/T (rs16944)	CC	26	22.6	>0.05	33	32.5	>0.05	47	46.6	>0.05
	CT	27	33.8		28	31.1		67	67.8	
	TT	16	12.6		9	7.5		25	24.6	

*IL-6*-174G/C (rs1800795)	GG	42	39.4	>0.05	43	44.2	>0.05	88	88.8	>0.05
	GC	21	26.3		24	23.7		43	41.4	
	CC	7	4.4		3	3.2		4	4.8	

*TNF-α*-1031T/C (rs1799964)	TT	50	50.3	>0.05	44	45.1	>0.05	82	80.5	>0.05
	TC	17	16.3		23	23.8		46	49.1	
	CC	1	1.3		3	3.1		9	7.5	

*TGFβ1*-509T/C (rs1800469)	TT	29	29.3	>0.05	30	32.9	>0.05	60	58.0	>0.05
	TC	32	31.3		36	30.2		57	61.0	
	CC	8	8.3		4	6.9		18	16.0	

**Table 3:** Genotype and allele frequencies of *IL-1β*-511C/T, *IL-6*-174G/C, *TNF-α*-1031T/C, and *TGFβ1*-509T/C gene polymorphisms in RSA patients with or without PCOS and controls.

Gene polymorphism		RSA with PCOS	RSA without PCOS	Control	RSA with PCOS *vs*. control			RSA without PCOS *vs*. control			RSA with PCOS *vs*. RSA without PCOS		
		*n* (%)	*n* (%)	*n* (%)	OR (95% CI)	*χ* ^2^	*p*	OR (95% CI)	*χ* ^2^	*p*	OR (95% CI)	*χ* ^2^	*p*
*IL-1β-*511C/T (rs16944)	*CC*	26 (37.7)	33 (47.9)	47 (33.8)	0.73 (0.35-1.47)	0.79	0.374	1.51 (3.43-0.66)	0.98	0.323	2.08 (0.85-5.09)	2.64	0.104
	*CT*	27 (39.1)	28 (39.4)	67 (48.2)	0.73 (0.37-1.40)	0.90	0.342	0.58 (0.31-1.07)	2.99	0.083	0.79 (0.38-1.65)	0.38	0.536
	*TT*	16 (23.2)	9 (12.7)	25 (18.0)	0.85 (0.46-1.54)	0.30	0.582	0.56 (0.31-0.99)	3.93	0.047^∗^	0.66 (0.33-1.29)	1.49	0.222
Allele	*C*	79 (57.3)	96 (67.6)	161 (57.9)	0.97 (0.64-1.47)	0.02	0.896	1.51 (0.99-2.31)	3.72	0.053	1.56 (0.95-2.53)	3.20	0.073
	*T*	59 (42.7)	46 (32.4)	117 (42.1)	1.03 (0.68-1.55)			0.66 (0.43-1.00)			0.64 (0.39-1.04)		
		*n* = 69	*n* = 70	*n* = 139									

*IL-6*-174G/C (rs1800795)	*GG*	42 (60.0)	43 (62.0)	88 (65.2)	0.28 (0.07-0.97)	4.50	0.033^∗^	0.69 (0.15-3.18)	0.23	0.634	2.52 (0.62-10.16)	1.78	0.181
	*GC*	21 (30.0)	24 (33.8)	43 (31.8)	0.28 (0.07-1.06)	3.81	0.050^∗^	0.74 (0.15-3.60)	0.14	0.712	2.67 (0.61-11.64)	1.78	0.181
	*CC*	7 (10.0)	3 (4.2)	4 (3.0)	1.25 (0.68-2.26)	0.53	0.464	1.15 (0.63-2.08)	0.21	0.647	0.92 (0.46-1.81)	0.06	0.810
Allele	*G*	105 (75.0)	112 (78.9)	219 (81.1)	0.70 (0.42-1.13)	2.08	0.149	0.87 (0.52-1.44)	0.30	0.587	0.87 (0.52-1.44)	0.60	0.440
	*C*	35 (25.0)	30 (21.1)	51 (18.9)	1.43 (0.87-2.33)			1.15 (0.69-1.90)			0.80 (0.46-1.40)		
		*n* = 70	*n* = 70	*n* = 135									

*TNF-α*-1031T/C (rs1799964)	*TT*	50 (73.5)	44 (62.5)	82 (59.9)	4.71 (0.58-37.97)	2.55	0.110	1.62 (0.42-6.17)	0.50	0.478	0.34 (0.03-3.38)	0.92	0.338
	*TC*	17 (25.0)	23 (33.3)	46 (33.6)	3.33 (0.39-28.25)	1.34	0.247	1.57 (0.38-6.32)	0.40	0.527	0.47 (0.04-4.91)	0.41	0.521
	*CC*	1 (1.5)	3 (4.2)	9 (6.5)	0.54 (0.28-1.01)	3.71	0.054	0.90 (0.49-1.60)	0.14	0.709	1.67 (0.81-3.42)	1.95	0.162
Allele	*T*	117 (86.0)	114 (79.2)	210 (76.6)	1.88 (1.07-3.28)	4.96	0.025^∗^	1.16 (0.71-1.89)	0.35	0.556	0.62 (0.32-1.15)	2.28	0.130
	*C*	19 (14.0)	30 (20.8)	64 (23.4)	0.53 (0.30-0.93)			0.86 (0.52-1.40)			1.62 (0.86-3.04)		
		*n* = 68	*n* = 70	*n* = 137									

*TGFβ1*-509T/C (rs1800469)	*TT*	29 (42.0)	30 (42.9)	60 (44.5)	1.17 (0.48-2.85)	0.12	0.724	2.54 (0.82-7.81)	2.79	0.094	2.16 (0.62-7.55)	1.52	0.217
	*TC*	32 (46.4)	36 (51.4)	57 (42.2)	1.26 (0.49-3.22)	0.24	0.625	2.84 (0.89-9.07)	3.30	0.069	2.25 (0.61-8.18)	1.57	0.210
	*CC*	8 (11.6)	4 (5.7)	18 (13.3)	1.10 (0.61-1.98)	0.11	0.742	1.07 (0.59-1.91)	0.05	0.828	0.97 (0.49-1.89)	0.01	0.921
Allele	*T*	90 (65.2)	96 (68.6)	177 (65.6)	0.99 (0.64-1.51)	0.00	0.945	1.15 (0.74-1.77)	0.38	0.539	1.16 (0.70-1.91)	0.35	0.552
	*C*	48 (34.8)	44 (31.4)	93 (34.4)	1.02 (0.66-1.56)			0.87 (0.56-1.34)			0.86 (0.52-1.41)		
		*n* = 69	*n* = 70	*n* = 135									

OR: odds ratio; CI: confidence interval; ^∗^significant difference.

## Data Availability

The data used to support the findings of this study are available from the corresponding author upon request.

## Additional Points

*Limitations*. (1) The study sample is confined to the Saudi nationality. The distribution of gene polymorphisms varies in different ethnicities. So, conclusions obtained in this study lack generalizability. (2) There is a relatively small sample size. The results need to be validated in larger samples in the future. (3) There is a lack of measures of the levels of the investigated cytokines. (4) The possibility of environmental risk factors was not included in the study, with the absence of environmental data.
